# COVID-19 Associated Choroidopathy

**DOI:** 10.3390/jcm10204686

**Published:** 2021-10-13

**Authors:** Youssef Abdelmassih, Georges Azar, Sophie Bonnin, Claire Scemama Timsit, Vivien Vasseur, Richard F. Spaide, Francine Behar-Cohen, Martine Mauget-Faysse

**Affiliations:** 1 Pediatric Ophthalmology and Retina Department, Rothschild Foundation Hospital, 75019 Paris, France; 2 Anterior Segment Department, Rothschild Foundation Hospital, 75019 Paris, France; georgesazar@hotmail.com; 3 Ophthalmology Department, Rothschild Foundation Hospital, 75019 Paris, France; sbonnin@for.paris (S.B.); cstimsit@for.paris (C.S.T.); 4 Clinical Research Department, Rothschild Foundation Hospital, 75019 Paris, France; vvasseur@for.paris (V.V.); mgfaysse@me.com (M.M.-F.); 5 Vitreous, Retina, Macula Consultants of New York, New York, NY 10022, USA; rickspaide@gmail.com; 6 Ophthalmology Department, Cochin Hospital, 75014 Paris, France; Francine.behar@gmail.com; 7 Centre de Recherche des Cordeliers, INSERM U1138, Team 17, Université de Paris, 75006 Paris, France

**Keywords:** COVID-19, SARS-CoV2, indocyanine green angiography, optical coherence tomography

## Abstract

The aim of the study is to report on the indocyanine green angiography (ICGA) and OCT findings in patients hospitalized for severe COVID infection. In this observational prospective monocentric cohort study, we included patients hospitalized for severe COVID infection. The main outcomes were ICGA and OCT findings. A total of 14 patients with a mean age of 58.2 ± 11.4 years and a male predominance (9/14 patients; 64%) were included. The main ICGA findings included hypofluorescent spots in 19 eyes (68%), intervortex shunts in 10 eyes (36%), and characteristic “hemangioma-like” lesions in five eyes (18%). “Hemangioma-like” lesions were both unique and unilateral, and showed no washout on the late phase of the angiogram. The main OCT findings included focal choroidal thickening in seven eyes (25%), caverns in six eyes (21%) and paracentral acute middle maculopathy lesions in one eye (4%). All patients hospitalized for severe COVID infection had anomalies on ICGA and OCT. Lesions to both retinal and choroidal vasculature were found. These anomalies could be secondary to vascular involvement related directly or indirectly to the SARS-CoV2 virus.

## 1. Introduction

In December 2019, the first cases of coronavirus disease 2019 (COVID-19) emerged in Wuhan province, China. The new coronavirus, named severe acute respiratory coronavirus 2 (SARS-CoV2), rapidly progressed to a pandemic [1]. By September 2021, more than 219,000,000 had become infected and more than 4,550,000 had died. Risk factors for morbidity and mortality of COVID-19 infection include coronary heart disease (CHD), hypertension, diabetes, male sex, smoking and obesity [2,3,4,5]. SARS-CoV2 penetrates the human cells by binding to the angiotensin-converting enzyme-2 (ACE-2) receptor which is present in the lungs and is highly expressed in the vascular endothelium [6]. SARS-CoV2 is responsible for multi-organ damages, either by direct virus attack or indirectly by inappropriate activation of the immune system and of both the complement and the coagulation cascade [7]. A high level of the von Willebrand Factor (vWF), most suggestive of endotheliopathy, is associated with a worse disease prognosis [8,9]. Recently, Yamaoka-Tojo reported on vascular endothelial glycocalyx damage secondary to COVID-19, resulting in systemic inflammatory microvascular endotheliopathy [10,11]. Indeed, the endothelial glycocalyx, composed of organized glycosaminoglycans produced and secreted by endothelial cells, tightly regulates innate immunity, inflammation, and coagulation. Its alteration induces exudation of fluid on the one hand and activation of coagulation cascade, thrombus formation and embolism on the other hand.

The prevalence of ocular involvement in COVID-19 is thought to be around 10% [12]. Casagrande et al. detected SARS-CoV-2 viral RNA in the retina of three deceased patients [13]. Clinically, cotton wool spots and hyper-reflective lesions at the level of the inner plexiform and ganglion cell layers have been described [14,15]. Choroidal involvement has also been reported but with discrepancy—choroidal vascular and stromal depletion on the one hand [16], and choroidal thickening in the macular region on the other hand [17]. Finally, some cases of atypical multifocal choroiditis and serpiginous choroiditis have recently been published [18,19].

Since SARS-CoV2 affects the microvasculature in many organs, the retinal and choroidal microvessels, which are easily accessible for observation and image acquisition, could be an open window on the general microvascular state of COVID-19 patients. However, choroidal and retinal vascular damages due to COVID infection, as well as their underlying pathophysiological mechanisms, are still poorly understood. The aim of our study is to report on the choroidal involvement imaged by indocyanine green angiography (ICGA) and optical coherence tomography (OCT) in a cohort of patients hospitalized for severe COVID infection, and to discuss potential pathophysiological mechanisms.

## 2. Methods and Materials

### 2.1. Study Design and Participants

In this observational prospective monocentric cohort study, we included patients hospitalized at the Rothschild Foundation Hospital, Paris, between 1 January and 30 June 2020, for severe COVID-19 infection. To be included in the study, patients needed to have an initial positive PCR test after oropharyngeal swab confirming SARS-CoV2 infection, chest-CT suggestive of COVID-19, or both. Patients also needed to be able to sit for a complete ophthalmological exam. The study was approved by the institutional review board (IRB) and the ethical committee (No. IDRCB: 2020-A01000-39) and adhered to the tenets of the Declaration of Helsinki. All patients (or their legal assignee when they were unable to sign for themselves) had to sign their informed consent explaining the aim of the study and the exams to undergo.

Medical records of included patients were studied and data concerning demographic characteristics and hospitalization were recorded. All patients underwent a complete ophthalmological exam including Early Treatment Diabetic Retinopathy Study (ETDRS) visual acuity (VA) measurement, slit-lamp examination, spectral-domain (SD) OCT B-scans, and ICGA. Images were acquired using Heidelberg Spectralis (Heidelberg Engineering, Germany). All the above examinations were performed after the patients were discharged from the intensive care unit (ICU) and once they were able to sit for OCT and ICGA. 

### 2.2. OCT Acquisition and Analysis

The OCT examination included macular line and volume scans. Additional line scans were performed during the ICGA when anomalies were observed. Mean central retinal thickness was automatically assessed using the device parameters, and choroidal thickness was manually measured using the caliper of the Heidelberg software on an enhanced depth imaging (EDI) scan. The same EDI scan was chosen to estimate the choroidal vascularity index (CVI), defined as the ratio of the luminal to choroidal area, by binarization of the subfoveal choroidal area using the ImageJ program (provided by the National Institutes of Health, Bethesda, MD, USA; http://imagej.nih.gov/ij/ accessed on 5 August 2021) as described by Sonoda et al. [20].

**Figure 1:**
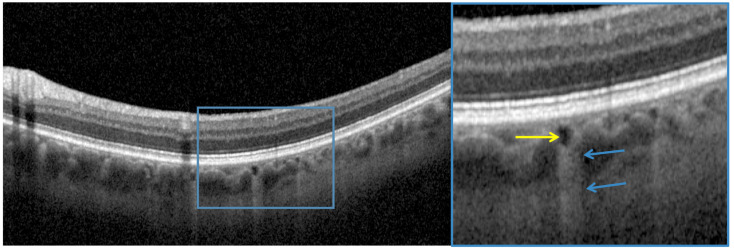
Optical coherence tomography showing a choroidal “cavern” (yellow arrow) defined as a focal area of internal hyporeflectivity with a characteristic hypertransmission signal underneath (blue arrows).

OCT anomalies were classified as the presence of: 1-choroidal caverns defined on OCT B-scans as focal areas of internal hyporeflectivity with typical features of outer contour angularity not corresponding to a choroidal vessel on ICGA (Figure 1) [21]; 2-focal choroidal thickening defined as an increase of ≥50 µm in choroidal thickness compared to the adjacent choroid [22], and 3-“pachyvessels”, defined, in this paper, as the presence of large vessels (>200 microns) with hyperreflective walls in Haller’s layer, underlying a thinned inner choroid, and present in the macular region [23].

### 2.3. ICGA Acquisition and Analysis

ICGA images were taken after the injection of 25 mg indocyanine (SERB LAB, Paris France) using a 60° camera, beginning in the very early phases followed by intermediate- and late-phase images of the posterior pole and the periphery. The images were analyzed by two retina specialists (SB and CS), and when found, discordance was settled by a third retina specialist (MMF). The early video-angiography was performed to appreciate watershed areas, general filling of the choroidal vessels and the presence of pulsatile shunts. 

**Figure 2:**
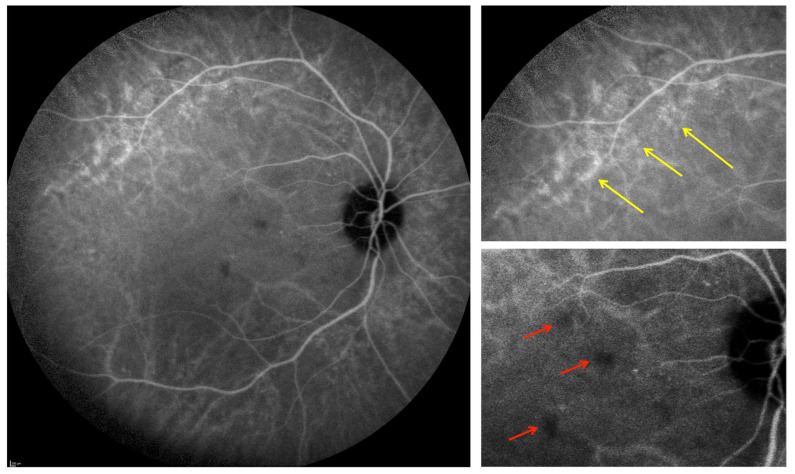
Intermediate phase of indocyanine green angiography showing dilated choroidal vessels with pinpoints (yellow arrows) and multiple hypofluorescent spots (red arrows).

**Figure 3:**
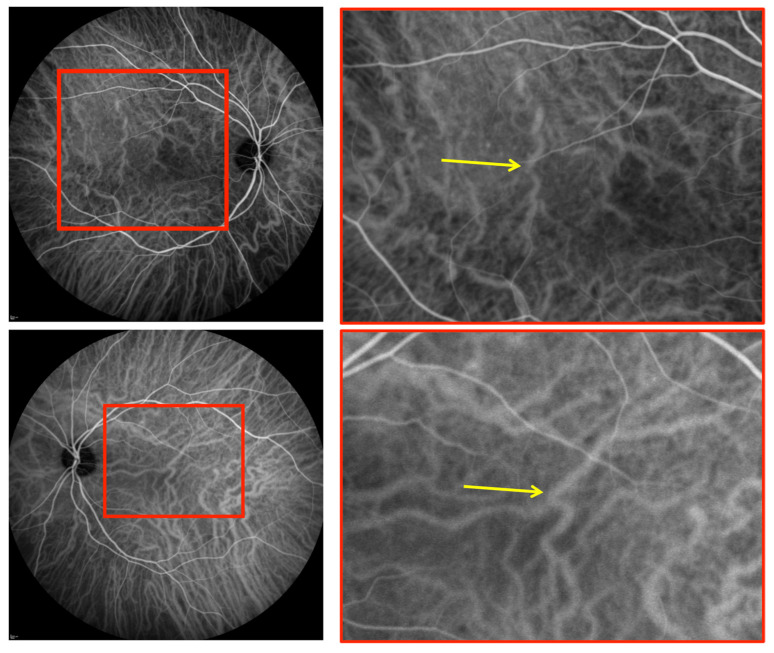
Indocyanine green angiography showing intervortex shunt (yellow arrows) between the superotemporal and inferotemporal vortex vein systems.

**Figure 4:**
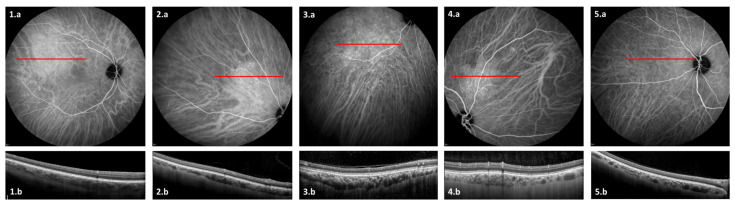
Intermediate phase of indocyanine green angiography (ICGA) (**1a**,**2a**,**3a**,**4a**,**5a**) and optical coherence tomography (OCT) (**1b**,**2b**,**3b**,**4b**,**5b**) of the 5 “hemangioma-like” lesions seen in our cohort. ICGA shows well-circumscribed hyperfluorescent choroidal area with pinpoints, which corresponds on OCT to zones of focal choroidal thickening with dilated choroidal vessels. These lesions correspond to patients’ numbers 3 (**1a**,**1b**), 7 (**2a**,**2b**), 10 (**3a**,**3b**), 12 (**4a**,**4b**), and 14 (**5a**,**5b**), respectively.

The ICGA images were analyzed to check for the presence of: 1-choroidal vessel leakage, defined as choroidal vascular hyperpermeability manifested by areas of hyperfluorescence, which are first seen during the early and intermediate phases of the angiogram and/or staining defined as marked fluorescence of dilated venous walls persistent until the late phase of the angiogram; 2-pinpoint leakage visible in the intermediate or late phases (Figure 2); 3-intervortex shunts defined as anastomotic connections between the superonasal, superotemporal, inferonasal and inferotemporal vortex vein systems (Figure 3) [24]; 4-hypofluorescent spots seen during the whole phase of the angiogram (Figure 2); as a well-circumscribed hyperfluorescent choroidal area with pin points on the ICGA intermediate phase, which corresponds to zones of dilated small choroidal vessels seen during the early phase and no washout in the late phase of the angiogram (Figure 4). 

### 2.4. Statistical Analysis

Data were collected and statistically analyzed using SPSS (version 22.0, Inc., Chicago, IL, USA). Descriptive statistics are reported as mean ± standard deviation for continuous variables and as percentage for categorical variables. Mann–Whitney U test was used to compare non-parametric continuous variables between groups.

## 3. Results

### 3.1. Demographic Characteristics of Patients

Fourteen patients (28 eyes) (with a mean age of 58.2 ± 11.4 years and a male predominance (9/14 patients; 64%)) were included. Table 1 reports on the baseline characteristics and medical records of included patients. Four patients had diabetes, of which two presented with background diabetic retinopathy. Eleven patients (79%) had acute respiratory distress syndrome (ARDS), of which eight (57%) were admitted at the ICU. Five patients (36%) presented with central nervous system (CNS) complications: myasthenia gravis (MG) decompensation, idiopathic cerebral hypertension (ICH), intraparenchymatous cerebral hematoma and two ischemic cerebrovascular accidents. Two patients had both ARDS and neurological complications. Since the indications of steroid therapy were not well established at the time of inclusion, only four patients (29%) received intravenous steroid therapy. Fibrinogen levels at presentation were reported for all but three patients; the mean level was 5.4 ± 2.6g/L. Fibrinogen levels were similar between patients with and without anomalies on OCT and ICGA. The mean BCVA was 82.2 ± 10.1 letters on the ETDRS scale, and the mean intraocular pressure (IOP) was 15.3 ± 3.2 mm Hg. One patient had a previous history of bilateral retinal artery occlusion and one patient had choroidal folds. Table 2 reports on the ophthalmological characteristics of included patients. Table 3 reports on the characteristics of each individual patient. 

### 3.2. OCT Findings

The mean central choroidal thickness was 265.3 ± 73.3 µm (67–432), the mean central retinal thickness was 273.4 ± 26.7 µm (183–320), and the mean CVI was 69.0 ± 2.7% (63–73). Only one eye had a choroidal thickness above 390 µm. Table 4 reports on choroidal thickness centrally and at 600 µm temporal and nasal to the fovea. Six eyes (21%) had at least one cavern and one eye (4%) had a paracentral acute middle maculopathy (PAMM) lesion. 

Interestingly, male predominance was found in patients with caverns (5 out of 6 patients (83%)). These eyes had higher CVI than eyes without caverns (71.2 ± 1.7% vs. 68.3 ± 2.7% *p* = 0.01) but similar choroidal thickness (275.1 ± 60.2 µm vs. 263.0 ± 78.1 µm *p* = 0.72). In eyes with caverns, five (83%) had leakage on ICGA, five (83%) had hypofluorescent spots, four (67%) had “hemangioma-like” lesions, and two (33%) had intervortex shunts.

### 3.3. ICGA Findings

With regard to ICGA signs, leakage and/or staining from choroidal vessels was seen in 20 eyes (71%), hypofluorescent spots in 19 eyes (68%), pinpoints in 15 eyes (54%), intervortex shunts in 10 eyes (36%), and “hemangioma-like” lesions in five eyes (18%). There was an association between caverns, hypofluorescent spots and “hemangioma-like” lesions in three eyes of three patients (two males and one female). None of these patients received systemic corticosteroids. OCT angiography (OCTA) scans were performed over accessible hypofluorescent spots and demonstrated diminished flow in the choriocapillaris area. 

Among the five eyes (five patients) with “hemangioma-like” lesions, four (80%) had leakage on ICGA, four (80%) had pachyvessels, and three (60%) had hypofluorescent spots. As described above with caverns, a male predominance was also found (4 out of 5 patients (80%)). Although choroidal thickness was not significantly higher in eyes that presented “hemangioma-like” lesions when compared to eyes that did not ((300.1 ± 76.4 µm vs. 258.4 ± 77.2 µm, respectively, (*p* = 0.19)), CVI was significantly increased in the former group ((72.0 ± 0.7% vs. 68.3 ± 2.5% *p* = 0.001)). When present, this lesion was always unique. Of note, in the contralateral eyes, four (80%) had leakage on ICGA and four (80%) had pachyvessels. Finally, compared to eyes with “hemangioma-like” lesions, the mean choroidal thickness in the contralateral eyes was slightly lower with a mean of 281.3 ± 49.1 µm (197–322), but this difference was not significant. 

## 4. Discussion

In this study, we performed both SD-OCT and ICGA on 14 patients (28 eyes) hospitalized for severe SARS-CoV2 to assess choroidal microvascular and structural anomalies potentially associated with the disease. 

On SD-OCT, six eyes (21%) presented with caverns defined as black irregular “walless” holes between choroidal vessels. These findings were observed in several pathologies such pachychoroid and geographic atrophy [21,25,26]. The exact nature of these caverns remains a subject of debate. Sakurada et al. found choroidal caverns in 52% of eyes with pachychoroid, especially in areas of choroidal vascular hyperpermeability on ICGA, suggesting that they could represent vessels with abnormal walls [21]. On the other hand, Dolz-Marco et al. defined caverns as a space filled with lipid globules [27]. Therefore, in the context of COVID-19, these caverns could correspond either to a new drainage route for the choroidal vessels through lymphatic system in case of overwhelmed “Starling principle” secondary to lesions inflicted on the choroidal endothelial cells via the alteration of their glycocalyx [28], or to lipid-filled spaces that may play an important role in the regulation of inflammatory processes. Further studies still need to be conducted in order to better understand the underlying process of choroidal caverns in the context of COVID-19. 

The vascular involvement was further confirmed by ICGA, which showed hypofluorescent lesions in 68% of eyes. The dark spots that persisted during the whole angiographic sequence represent choriocapillaris hypoperfusion, as confirmed by OCTA. These ischemic spots could be secondary SARS-CoV-2-induced damages which include direct viral-induced cell death, inappropriate inflammatory reaction with major vascular inflammation and neutrophil infiltration [29], and finally thromboembolic events demonstrated by the presence of prolonged activated partial thromboplastin (aPTT) and prothrombin time (PT), elevated D-dimer, thrombocytopenia, as well as the presence of hyaline thrombi as found in pulmonary and cardiac microvessels [30,31]. In fact, some patients in our series presented signs of systemic thromboembolism, such as pulmonary embolism and cerebrovascular accident as previously described. The alteration of this coagulation cascade seen in COVID-19 is thought to be correlated with endothelial glycocalyx damages [10,11]. In fact, the glycocalyx protects the endothelium from oxidative and sheer stress, regulates vascular permeability, microvascular tonus, and leukocyte adhesion [32,33]. Therefore, its disruption and degradation, identified by high circulating levels of syndecan-1, hyaluronic acid and heparan sulfate in COVID-19 patients, contributes to an increased risk of thrombosis [34,35].

Additional ischemic lesions including PAMM were found in one patient. This finding aligns with the results of other authors in the literature. In fact, Pereira et al. described ischemic pattern lesions defined as cotton wool spots and retinal sectorial pallor in 22% of patients [36]. Virgo and Mohamed reported on two cases of acute macular neuroretinopathy (AMN) [37]. Finally, Turker et al. described the effect of COVID-19 on the retinal capillary plexus and choriocapillaris using OCTA [38]. 

Leakage and staining from choroidal vessels were observed in 71% of eyes. In our opinion, this leakage is also linked to the SARS-related damage to the glycocalyx structure, which leads to increased choroidal vascular permeability, leading to interstitial fluid shifts and edema. This can be similar to COVID-19-induced ARDS mechanisms, where glycocalyx alteration and shedding increase alveolar barrier permeability by disrupting tight-junction proteins [39]. 

Many reports have studied the effect of systemic diseases and infections on retinal and choroidal perfusion, and the effect of blood oxygenation (oxygen and carbon dioxide) on retinal and choroidal blood flow [40,41,42]. In addition, local inflammation secondary to COVID-19 could result in the alteration of the effect of the autonomic nervous system and the destruction of the vascular smooth muscle cells in the choroid [43,44]. These alterations may induce postcapillary venules formation, which could result in the creation of abnormal medium-sized vessels communication in Sattler’s and Haller’s layers, explaining the intervortex communication found in 36% of our patients [45].

Unlike caverns, hypofluorescent spots, intervortex shunts and leakage may also be seen in cases of non-COVID-19 patients; a striking choroidal sign, the “hemangioma-like” lesion, is a specific sign not previously described. This lesion may be a consequence of venous overload and leakage downstream of the microthrombosis. Interestingly, eyes presenting with “hemangioma-like” lesions had a higher CVI compared to contralateral eyes of the same patient and to eyes without this lesion, which suggests the role of vessel dilation in this particular finding. Another mechanism could also involve a deregulation in the choroidal blood flow that could result in vascular remodeling. This choroidal blood flow deregulation could be due to the alteration of the autonomous nervous system secondary to COVID-19 infection [43,44]. Of note, unlike real hemangioma lesions that usually present with RPE bulging and late washout on ICG, none of those “hemangioma-like” lesions presented these two characteristics. 

Our study has several limitations. They include the small number of patients, the absence of prior ICGA to demonstrate the temporal relationship between the anomalies and the COVID-19 infection, and the absence of a control group. Furthermore, since fluorescein angiography was not performed in these patients due to the risk of allergic reactions and the general health of these fragile patients, no conclusion could be reached on the status of retinal vasculature such as ischemia and diffusion.

## 5. Conclusions

Choroidal anomalies seen on both ICGA and OCT were frequently observed in patients hospitalized for severe COVID-19 infection. Anomalies include caverns, hypofluorescent spots, leakage, intervortex shunts, and finally “hemangioma-like” lesions, the nature and origin of which are yet to be determined. This particular sign appears as a well-circumscribed zone of hyperfluorescence in the early phases of the angiogram and does not show washout of the dye in the late phase. Although frequent in patients with COVID-19, we cannot assume with certainty that these anomalies were caused by COVID-19. Future studies including a control group would be necessary to attribute these abnormalities to COVID-19 infection. 

## Figures and Tables

**Table 1:** Baseline and medical characteristics of included patients. ARDS: acute respiratory distress syndrome; CT: computed tomography; PCR: polymerase chain reaction; SD: standard deviation.

Variables	
Age in year (mean ± SD)	58.2 ± 11.4
Gender	
- Male - Female	9 (64%) 5 (36%)
COVID-19 diagnosis method	
- PCR - CT-scan - PCR + CT-scan	4 (29%) 6 (43%) 4 (29%)
Causes of hospitalization	
ARDS Neurologic	11 (79%) 5 (36%)
Oxygen need	10 (71%)
Intensive care unit admission	8 (57%)
Corticosteroid use	5 (29%)

**Table 2:** Ophthalmological characteristics of included patients. ETDRS: Early Treatment Diabetic Retinopathy Study; ICGA: indocyanine green angiography; IOP: intraocular pressure; OCT: optical coherence tomography; SD: standard deviation.

Variables	
Visual acuity in ETDRS letters (mean ± SD)	82.2 ± 10.1
IOP (mean ± SD)	15.3 ± 3.2
ICGA anomalies	Number of eyes (%)
Vessel leakage and/or staining Hypofluorescent spots Pintpoint leakage Intervortex shunts “Hemangioma-like” lesion	20 (71%) 19 (68%) 15 (54%) 10 (36%) 5 (18%)
OCT anomalies	Number of eyes (%)
Pachyvessels Focal choroidal thickening Caverns	25 (89%) 7 (25%) 6 (21%)
Choroidal thickness in μm (mean ± SD (range))	265.3 ± 73.3 (67–432)
Central macular thickness in μm (mean ± SD (range))	273.4 ± 26.7 (183–320)

**Table 3:** Medical, indocyanine green angiography (ICGA) and optical coherence tomography (OCT) characteristics of each patient. ARDS: acute respiratory distress syndrome; ARF: acute renal failure; CSR: central serous retinopathy; CVA: cerebrovascular accident; DKA: diabetic ketoacidosis; HF: heart failure; NA: not applicable; ND: not determined; PAMM: paracentral acute middle maculopathy; PE: pulmonary embolism.

							ICG Signs					OCT Signs			Other Anomalies
Patient Number	Sex	Age in Years	Hospitalization Reasons	ARDS Stage	Intensive Care Unit Admission	Corticosteroid Use	Hypofluorescent Spots	“Hemangioma-Like” Lesion	Intervortex Shunts	Pintpoint Leakage	Vessel Leakage and/or Staining	Caverns	Pachyvessels	Focal Choroidal Thickness	
1	Male	64	ARDS with myasthenia decompensation	ND	No	Yes	Yes	No	Yes	Yes	No	No	Yes	Yes	Choroidal folds
2	Male	48	ARDS with DKA and ARF	Moderate	Yes	No	No	No	No	Yes	Yes	No	Yes	No	
3	Male	70	ARDS with PE and HF	Severe	Yes	No	Yes	Yes	No	Yes	Yes	Yes	No	No	Retinal atrophy
4	Female	63	ARDS with DKA	ND	No	No	Yes	No	No	Yes	Yes	No	Yes	Yes	
5	Male	65	ARDS	Severe	Yes	Yes	Yes	No	Yes	Yes	No	Yes	Yes	No	
6	Female	27	Idiopathic cerebral hypertension	NA	No	No	Yes	No	No	No	Yes	No	Yes	No	Papillary edema
7	Male	66	ARDS	ND	Yes	No	Yes	Yes	Yes	Yes	Yes	Yes	Yes	Yes	
8	Male	55	ARDS	Severe	Yes	Yes	Yes	No	Yes	Yes	Yes	No	Yes	Yes	
9	Male	74	Ischemic CVA	NA	No	No	Yes	No	No	No	Yes	No	Yes	No	Pseudodrusen
10	Male	59	Ischemic CVA	NA	No	No	Yes	Yes	Yes	Yes	Yes	Yes	Yes	No	
11	Female	65	ARDS	Severe	Yes	Yes	Yes	No	Yes	Yes	Yes	No	Yes	No	PAMM
12	Male	61	ARDS	Severe	Yes	No	No	Yes	Yes	Yes	No	No	Yes	Yes	CSR
13	Female	55	ARDS with cerebral thrombophlebitis, intraparenchymatous hematoma and febrile confusion	Moderate	No	No	No	No	Yes	No	Yes	No	Yes	Yes	
14	Female	49	ARDS	Severe	Yes	No	Yes	Yes	No	Yes	Yes	Yes	Yes	Yes	

**Table 4:** Choroidal thickness in relation to spherical equivalence (SE) of each included patient. CVI: choroidal vascularity index; D: diopter.

Patient Number	SE in D	Foveal Choroidal Thickness in µm	Choroidal Thickness 600 µm Nasal to the Fovea in µm	Choroidal Thickness 600 µm Temporal to the Fovea in µm	CVI in %
1	3	190	173	213	70
	3.5	200	186	158	71
2	0	337	324	337	69
	0	304	317	317	69
3	−1	325	325	303	73
	−1	290	269	255	68
4	0	268	235	308	70
	0	272	291	269	67
5	0	232	220	241	69
	0	242	234	201	71
6	−1.25	351	338	386	68
	−1	432	407	392	70
7	0	291	282	304	72
	0	197	158	204	69
8	0	302	282	282	70
	0	287	290	311	73
9	2.5	185	117	145	63
	2.75	67	90	41	66
10	1.25	356	386	330	72
	2	294	382	207	67
11	1.25	156	165	172	67
	1.5	176	179	200	65
12	3.5	322	344	234	64
	2.75	280	345	325	71
13	−3.5	274	189	291	66
	−2.75	248	207	159	66
14	5.25	248	276	207	72
	7	302	276	310	72

## Data Availability

All data are available upon reasonnable request due to patient confidentiality.
